# Tumor Necrosis Factor Induces Developmental Stage-Dependent Structural Changes in the Immature Small Intestine

**DOI:** 10.1155/2014/852378

**Published:** 2014-08-27

**Authors:** Kathryn S. Brown, Huiyu Gong, Mark R. Frey, Brock Pope, Matthew Golden, Katerina Martin, Mitchel Obey, Steven J. McElroy

**Affiliations:** ^1^Department of Pediatrics, University of Iowa, Iowa City, IA 52242, USA; ^2^Departments of Pediatrics and Biochemistry and Molecular Biology, University of Southern California Keck School of Medicine and The Saban Research Institute at Children's Hospital Los Angeles, Los Angeles, CA 90027, USA

## Abstract

*Background*. Premature infants are commonly subject to intestinal inflammation. Since the human small intestine does not reach maturity until term gestation, premature infants have a unique challenge, as either acute or chronic inflammation may alter the normal development of the intestinal tract. Tumor necrosis factor (TNF) has been shown to acutely alter goblet cell numbers and villus length in adult mice. In this study we tested the effects of TNF on villus architecture and epithelial cells at different stages of development of the immature small intestine.* Methods*. To examine the effects of TNF-induced inflammation, we injected acute, brief, or chronic exposures of TNF in neonatal and juvenile mice.* Results*. TNF induced significant villus blunting through a TNF receptor-1 (TNFR1) mediated mechanism, leading to loss of villus area. This response to TNFR1 signaling was altered during intestinal development, despite constant TNFR1 protein expression. Acute TNF-mediated signaling also significantly decreased Paneth cells.* Conclusions*. Taken together, the morphologic changes caused by TNF provide insight as to the effects of inflammation on the developing intestinal tract. Additionally, they suggest a mechanism which, coupled with an immature immune system, may help to explain the unique susceptibility of the immature intestine to inflammatory diseases such as NEC.

## 1. Introduction

Intestinal inflammation is a common occurrence for premature infants. After a relatively protective intrauterine environment, the newborn is abruptly exposed to nonmaternal antigens and abnormal bacterial flora in the intensive care setting [1]. Even normal food sources for infants can induce inflammation in immature intestine. Exposure to formula has been shown to increase proinflammatory cytokines in the immature intestine [2], and breast milk from mothers exposed to a Western diet has been shown to contain increased amounts of long chain and saturated fatty acids that induce inflammation in the immature intestine [3]. While these data indicate that diets of premature infants put them at risk for chronic gut inflammation, the effects of chronic inflammation on the developing intestine are not fully understood. Notably, enteral feeding is one of the strongest risk factors for the development of necrotizing enterocolitis (NEC) [4], leading to questions regarding the role that feeding-induced chronic intestinal inflammation may play in the induction and pathophysiology of the disease.

NEC is predominantly a disease of premature infants, primarily affecting infants born at the shortest gestations [4, 5]. The incidence of NEC is the highest between the ages of 28 and 32 weeks relative gestation regardless of when an infant is born, suggesting that changes in gut development regulate susceptibility [6, 7]. A key component of NEC is exaggerated inflammation [8–10] mediated by potent inflammatory cytokines such as tumor necrosis factor (TNF) and platelet activating factor (PAF) [11]. In human studies, newborn infants who developed NEC expressed significantly higher serum TNF levels compared to controls [11], and in animal models of NEC, animals treated with anti-TNF antibodies have lower incidence and severity of disease [12]. Our lab recently reported that significant developmental-stage dependent changes can be seen in intestinal goblet cell mucus secretion in the murine ileum eight hours following TNF exposure [13]. Furthermore, acute TNF exposure mediates the early stages of tissue damage seen in* Salmonella* infection of the intestinal tract [14] and can induce decreases in villus height in burn patients [15]. However, the specific role of TNF in the pathophysiology of NEC is not completely known and the mechanisms behind the initiation of inflammatory signaling represent a major gap in our understanding.

Considering that (1) susceptibility to NEC appears to be developmentally-dependent, (2) premature infants have frequent exposure to intestinal inflammation, and (3) TNF induced inflammation can have developmental dependent effects on the intestinal epithelium, we tested whether TNF induces other developmentally dependent changes in the small intestine. To better understand how different exposures of inflammation would affect the developing intestinal tract, we examined components of intestinal architecture following several different types of exposure to TNF. In the following studies, we show that both acute and chronic exposure to TNF have developmental-stage dependent effects on the developing small intestine.

## 2. Materials and Methods

### 2.1. Mice and TNF Injections

C57BL/6J, TNFR1^−/−^, TNFR2^−/−^, and TNFR1^−/−^2^−/−^ mice were purchased from The Jackson Laboratory. CD-1 mice were purchased from Charles River Laboratories. All genetically modified mice were maintained on a C57BL/6J background and C57BL/6J was used as wild type comparisons. For acute TNF experiments, C57BL/6J or CD-1 mice were injected intraperitoneally at the indicated ages with 0.5 *μ*g/gbw TNF (PeproTech) and maintained for 8 hours separated from mothers and without feeds at 33°C in a humidified incubator (Petiatric.com). The distal 1/3 of the small intestine was harvested and isolated for ileal samples. Tissues were fixed by snap freezing or Carnoy solution; tissues prepared for immunohistochemistry were paraffin-embedded and sectioned at 5 *μ*m. For chronic TNF experiments, CD-1 mice were given weekly intraperitoneal injections with 0.5 *μ*g/gbw TNF. Injections began at age P7 and ended one week prior to euthanasia. During injections, mice were housed under normal conditions at the animal care facility at the University of Iowa. On the final day of experimentation, tissue was harvested as described above. All animal experiments were performed according to protocols approved by the Institutional Animal Care and Use Committee at The University of Iowa.

### 2.2. Villus Morphometry and Cell Counts

Ileal sections were stained with H&E (Sigma-Aldrich). In an attempt to minimize sectioning variability, all sections were obtained from the center of the intestinal sample and only areas with full villi were included. In each sample used for measurement, at least 3 areas were counted to minimize sectioning variances. Measurements were obtained at each timepoint from at least five separate animals. A total of at least 20 consecutive villi from 3 separate areas per animal were analyzed in each experimental group. Villus length and surface area measurements were taken with a 10x objective (100x total magnification) from the tip of the villus to the entrance of the crypt opening. Villus length was measured by drawing a bisecting line through the center of the villi. Surface area was measured by drawing a line around the exterior of each structure. Villus epithelial cells were counted with a 60x objective (600x total magnification) from the entrance of the crypt opening to the beginning of the curve at the villus tip. Intestinal sections from at least five animals for a total of at least 70 villi per animal were analyzed for each experimental group. To detect Paneth cells, slides were stained with Alcian Blue/Periodic Acid Schiff stain (Sigma-Aldrich). Cells were quantified with a 60x objective (600x total magnification) by a single blinded investigator. Intestinal sections from at least five animals were analyzed for each experimental group and at least 100 crypts were counted per animal. All data were obtained using a Nikon NiU microscope using Nikon Elements software (Nikon).

### 2.3. Cell Lysates, PCR, and Western Blotting

Ileal samples were homogenized using a TissueLyser LT (Qiagen), then cleared, and boiled as previously described [16]. Proteins were separated by SDS-PAGE and transferred to nitrocellulose membranes. Membranes were blocked for 1 h in Tris-buffered saline with 0.05% Tween 20 (TBST) and 5% nonfat dry milk, incubated with anti-TNFR1 primary antibody (Santa Cruz Biotechnology) overnight at 4°C, and incubated with secondary antibody (Cell Signaling) for 45 minutes. Horseradish peroxidase was detected with the Western Lightning enhanced chemiluminescence kit (PerkinElmer Life Sciences). For mRNA quantification, ileal samples were homogenized as above and RNA was isolated using RNeasy Plus Mini Kit (Qiagen) according to manufacturer's directions. RNA concentration and quality were determined using a NanoDrop 1000 Spectrophotometer (Thermo Fisher Scientific). First-strand cDNA was synthesized using High-Capacity cDNA Reverse Transcription Kit with RNase Inhibitor (Life Technologies). Quantitative real-time reverse transcription-polymerase chain reaction (qRT-PCR) was performed using Taqman Fast Universal PCR Master Mix (2X) (Life Technologies) and Taqman Gene Expression Assays for TNFR1 primers (Life Technologies). qRT-PCR reactions were run in a C1000 Thermal Cycler (Eppendorf) and using the CFX96 Real-Time PCR Detection System (Bio-Rad). 10 ng of cDNA was used per reaction. The threshold cycle (CT) value for each well was obtained by using the instrument's software. Fold change in gene expression was determined by normalizing gene expression to *β*-actin in each sample. The 2ΔΔ-CT method was used to compare gene expression levels between samples.

### 2.4. Apoptosis Assays

Rates of apoptosis on histological specimens were determined by in situ oligo ligation DNA fragmentation assay (ISOL; Millipore), as we have previously reported [17].

### 2.5. Replicates and Statistical Analysis

All data are representative of at least three independent experiments. Statistical significance of differences was assessed with one-way analysis of variance (ANOVA). Post-test comparisons between groups were made using Holm-Sidak's multiple comparisons test. All statistics were performed using Prism software (Graph Pad). Minimum level of significance was set at <0.05 and error bars designate standard error of the mean.

## 3. Results

### 3.1. Ileal Villus Length Increases Significantly during Specific Stages of Development

**Figure 1:**
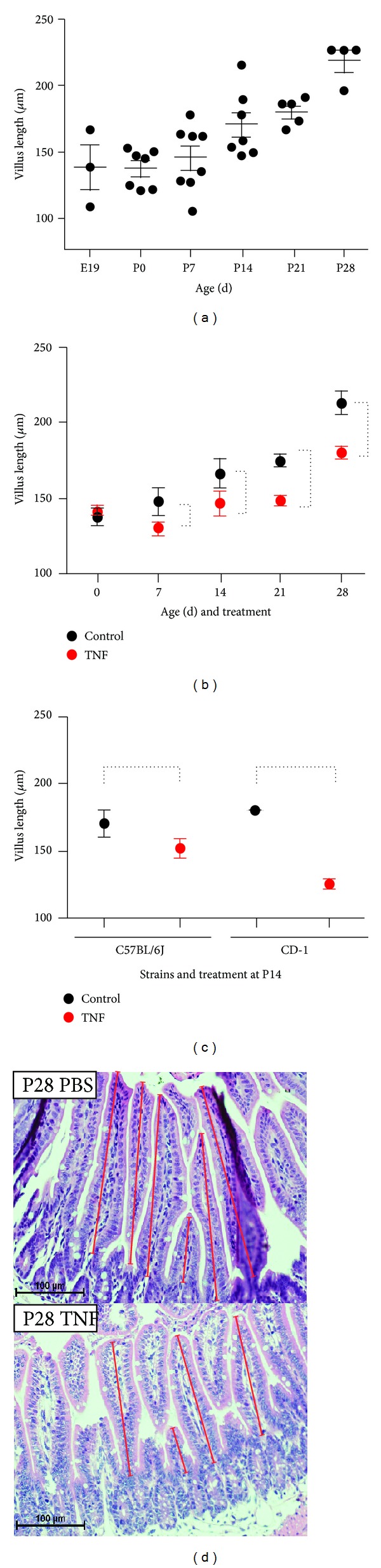
Acute TNF blunts ileal villus length at specific stages of development. (a) The ileal segment of the small intestine was harvested from mice at indicated ages and villus length was microscopically measured. Each dot represents one animal's average villus length which was generated by averaging at least 20 consecutive villi from 3 separate areas per animal. (b) Mice were treated with TNF at indicated ages and villus length was determined (red, TNF; black, control; *n* = 5 for each point; *P* < 0.0002, significance is denoted by dashed lines). (c) Small intestinal villus length was measured at P14 following TNF treatment and compared to controls in both C57BL6 and CD1 mice (*n* = 5; *P* < 0.003 for both strains). (d) Representative examples of histology sections at different developmental stages.

We have previously shown that goblet cell numbers in the murine ileum increase in a developmental-dependent manner during the first four weeks of life [13]. To determine if ileal height developed in a similar pattern, we measured the length of ileal villi at various stages of development throughout the first four weeks of life of the mouse. Ileal villus length increased steadily during development (Figure 1(a)). Birth did not affect villus length, as there was no difference between E19 and P0 mice.

### 3.2. Acute TNF Blunts Ileal Villus Length at Specific Stages of Development

We have previously shown that TNF has an age-dependent effect on intestinal mucus production and secretion [13]; however, the effects of TNF on the developing intestinal morphology in the immature intestine remain unknown. To test these effects, we examined the ileum eight hours after an intraperitoneal injection with TNF. This time point was based on our earlier studies of TNF exposure on goblet cell numbers in immature intestine [13, 16]. Villus lengths of treated mice were compared to age-matched controls. TNF induced significant villus blunting at P7, P14, P21, and P28 (Figure 1(b)). In contrast, P0 pups were protected from this effect, suggesting either the presence of a protective signaling pathway or decreased responsiveness to TNF at this stage of development. To determine if TNF-induced villus blunting was specific to the C57Bl/6J strain, we repeated this experiment in P14 CD-1 mice and compared these data to the results seen in C57Bl/6J mice from Figure 1(b). TNF significantly blunted ileal villi in CD-1 mice as well (Figure 1(c)). Our data are thus consistent with a strain-independent effect, though it should be noted that showing true strain independence would require experiments with many more lines, which is beyond the scope of this study.

### 3.3. TNF-Induced Villus Blunting Is Mediated by Reduction in Villus Surface Area and Epithelial Cell Loss

**Figure 2:**
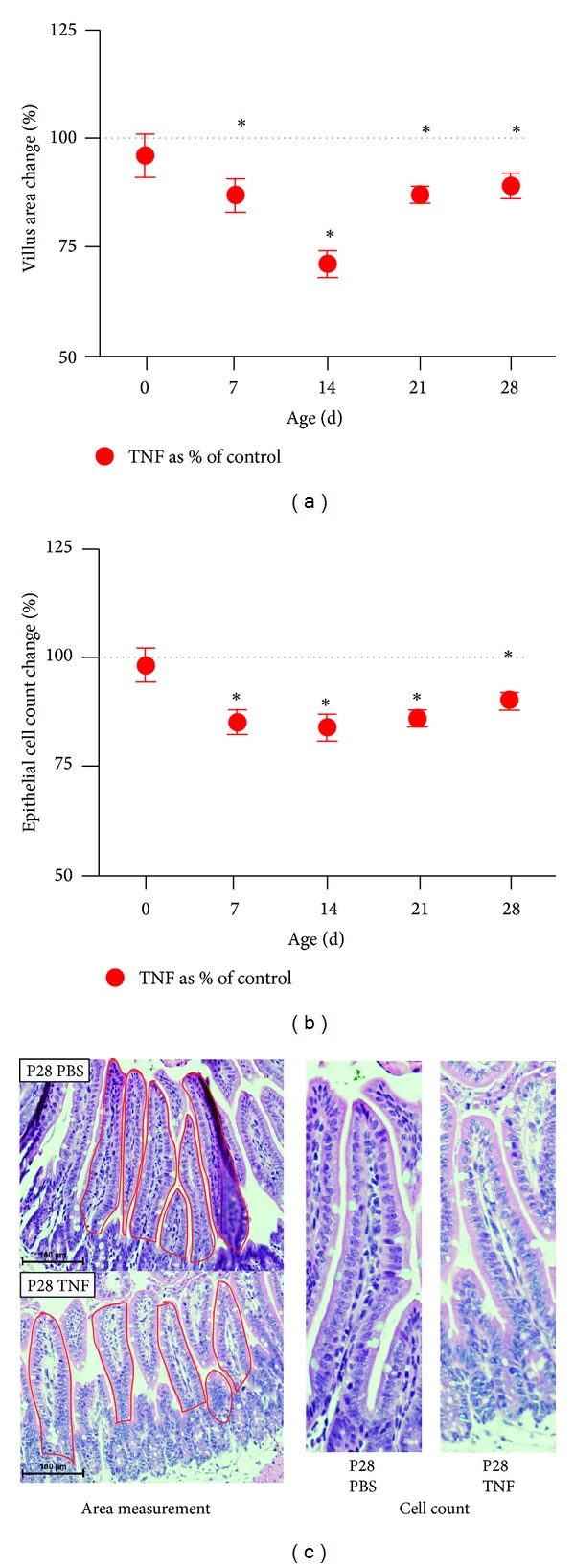
Acute TNF induces loss of ileal villus area and epithelial cell mass during development. Ileal segments of the small intestine were harvested from control and TNF-treated mice at indicated ages. (a) Villus areas were determined by tracing perimeters in Nikon Elements software; (b) epithelial cell numbers were microscopically quantified. Data are shown as the TNF-induced change versus controls (which are set to 100%). Significant differences from controls at each equivalent age are denoted by an asterisk (*n* = 5; *P* < 0.0002). (c) Representative tissue samples are shown.

To further characterize TNF-induced changes in intestinal morphology, we quantified the epithelial cells lining villus cross-sections. Similar to the effects seen on villus length, the number of villus epithelial cells was not affected by TNF at birth. However, in the subsequent 4 weeks, TNF induced a significant decrease in total epithelial cells compared to controls (Figure 2(b)). We next measured the average villus area of mice treated with TNF compared to controls. In control animals, villus area significantly decreased in the first week of life, followed by significant increases during the second, third, and fourth weeks of life. TNF treatment again had no significant effect at birth but induced significant reductions in villus surface area at later ages (*P* < 0.0001) (Figure 2(a)).

### 3.4. TNF Induces Increased Epithelial Apoptosis in Immature Intestine

**Figure 3:**
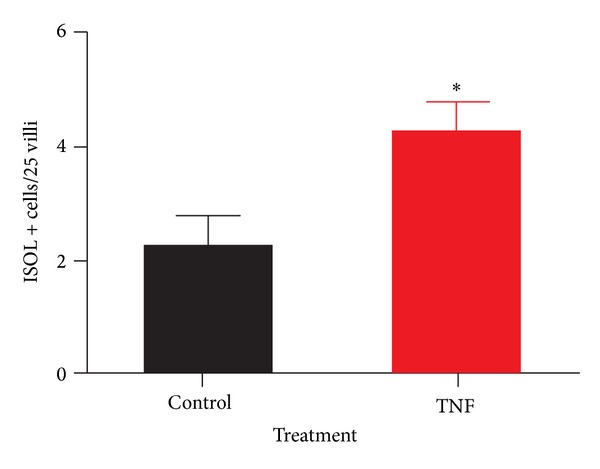
TNF induces increased villus apoptosis in immature intestine. P14 small intestinal samples were stained by in situ oligo ligation (ISOL) for DNA fragmentation and compared to controls. Positive events per 25 villi were counted microscopically. A significant increase in events was observed in the TNF-treated group (*n* = 5; *P* = 0.03).

To test if TNF-induced loss of villus height, surface area, and epithelial cell mass could be due to induction of apoptosis, histological samples from P14 mice treated with TNF were stained by in situ oligo ligation (ISOL) to detect DNA fragmentation and compared to controls. Mice exposed to acute TNF had significantly more apoptosis than age-matched controls (*P* = 0.03) (Figure 3).

### 3.5. TNF-Induced Villus Blunting Requires TNFR1

**Figure 4:**
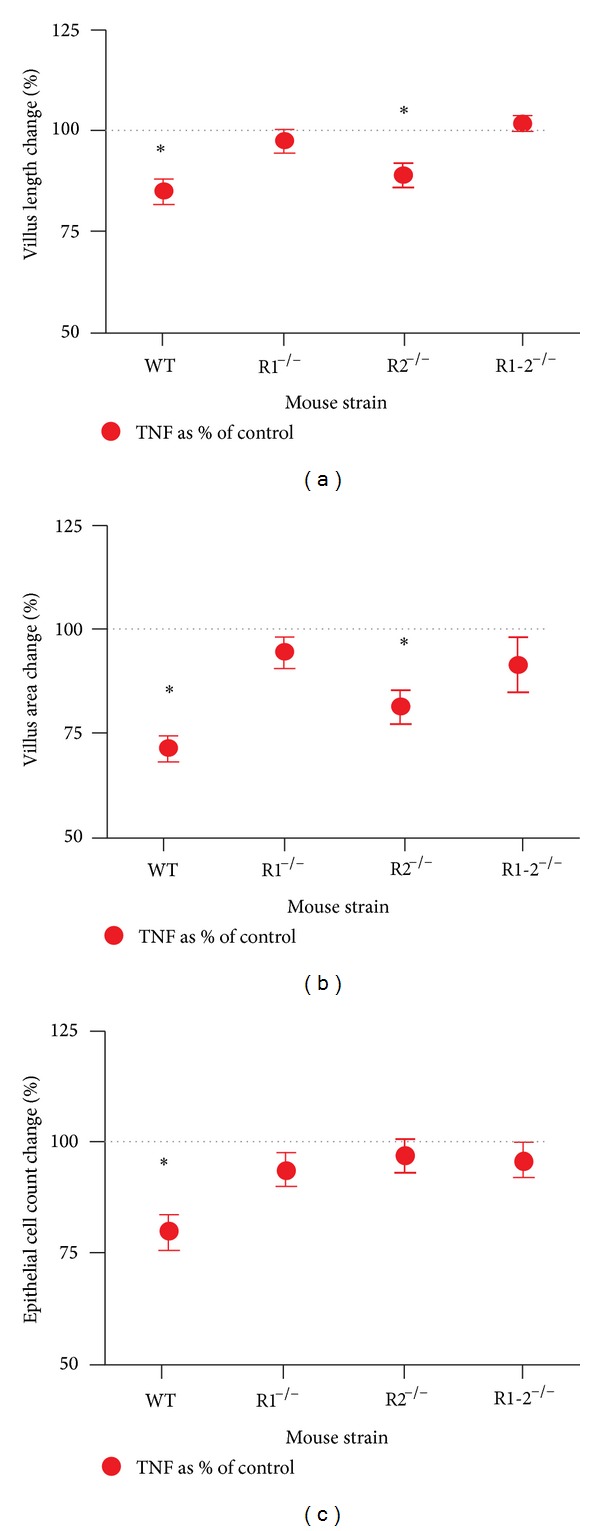
TNFR1 is required for TNF-induced ileal villus blunting and villus area loss. Small intestinal villus length, area, and epithelial cell counts were measured as above in mice lacking one or both TNFRs (R1^−/−^, R2^−/−^, or R1-2^−/−^). Mice treated with TNF were measured and compared to controls of the same strain. Data are shown as the difference from controls, which are set to 100%. Significant differences (*P* < 0.002) from controls in TNF treatment are denoted by an asterisk.

We have previously shown that TNF can affect both intestinal epithelial cells and ileal goblet cells through TNF receptor-1 (TNFR1) signaling [13, 16]. To determine if the TNF-dependent effects on villus length, area, and epithelial cell mass were also dependent on TNFR1, P14 TNFR1^−/−^, TNFR2^−/−^, and TNFR1^−/−^2^−/−^ mice were given intraperitoneal injections of TNF and their ileal villus measurements were compared to knockout controls. TNF induced villus blunting in wild type and TNFR2^−/−^ mice, but not mice lacking TNFR1. This demonstrates a requirement for TNFR1, and no role for TNFR2. Similarly, TNF treatment induced loss of villus area only in wild type and TNFR2^−/−^ mice, again demonstrating a requirement for the presence of TNFR1. However, only wild type mice showed a significant loss in epithelial cell number (Figure 4).

### 3.6. Ileal Expression of TNFR1 Is Constant during Development

**Figure 5:**
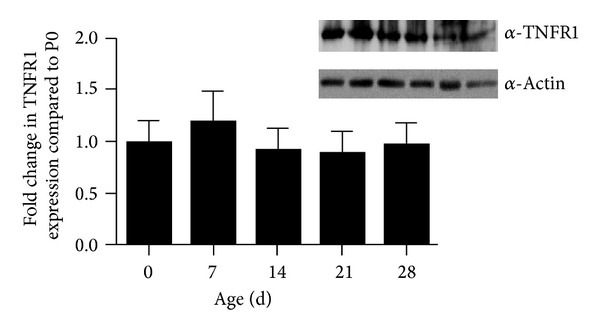
TNFR1 expression in small intestine is constant during development. TNFR1 protein expression levels were tested by western blot in small intestinal homogenates from P0 to P28 mice. A representative western blot is shown. The graph shows the average TNFR1 expression at noted ages (*n* = 5 animals/group; expression normalized to actin; all ages compared to P0). No significant difference was detected between any ages.

Our findings indicate that the degree of TNF-induced villus blunting varies depending on the stage of development. To test whether these effects were due to differences in ileal maturity, rather than ontogenic differences in TNFR1 expression, ileal tissue was harvested from wild type mice at various ages and examined by Western blot analysis for expression of TNFR1. No significant differences in TNFR1 expression were detected during development (Figure 5). This finding is important, as it demonstrates a developmental stage-dependent change in downstream TNFR1 effectors that alter response without changing receptor expression.

### 3.7. Chronic TNF Exposure Induces Villus Blunting in a Dose Dependent Fashion

**Figure 6:**
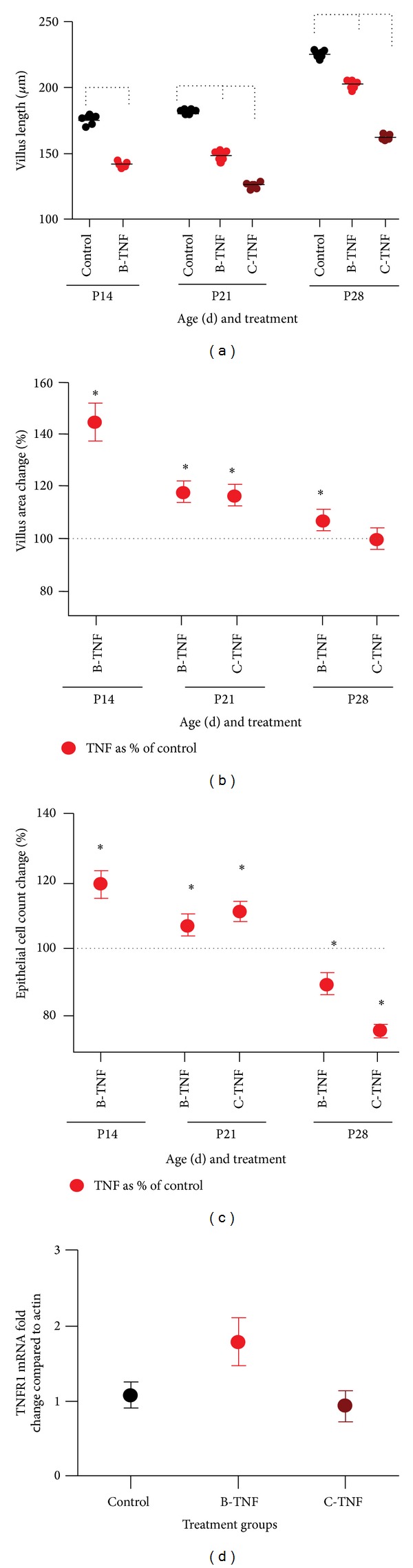
Chronic TNF treatment has exposure-dependent effects on small intestinal architecture. Mice were treated with TNF one week prior to euthanasia (brief exposure designated as B-TNF), or weekly starting on P7 until one week prior to euthanasia (chronic exposure designated as C-TNF). Small intestine was harvested and examined for (a) villus height, (b) villus area, and (c) epithelial cell counts as described above. For all groups, *n* = 5. Brackets in (a) indicate *P* < 0.0001. Asterisks in (b) and (c) indicate *P* < 0.0001 in TNF versus control. (d) To determine if chronic exposure to TNF impacts TNFR1 levels, mRNA levels of TNFR1 were quantified in tissues of P21 mice treated with B-TNF and C-TNF compared to controls. No significant differences in TNFR1 levels were observed.

Since our data demonstrate a developmentally dependent effect of acute TNF on the architecture of the ileum, we investigated the effects of chronic exposure. To accomplish this, mice were treated with TNF once a week beginning on P7 until one week prior to euthanasia. In this fashion, we developed three groups of mice: control mice, mice exposed to a single dose of TNF one week prior to examination (brief exposure, or B-TNF), and mice exposed to weekly TNF treatments beginning on P7, and ending with the last dose occurring one week prior to euthanasia (chronic exposure, or C-TNF). Using this methodology, we examined the ilea of mice from each group for villus length. Similar to the acute exposure, mice exposed to chronic TNF showed a significant loss in villus length. Mice treated with brief exposure (B-TNF) had significantly shorter villi than controls, and mice treated with chronic exposure (C-TNF) had significantly shorter villi than those with brief exposure (Figure 6(a)). To determine if chronic exposure to TNF would impact the levels of TNFR1, we measured levels of TNFR1 mRNA in tissues of P21 mice treated with B-TNF and C-TNF compared to controls. We chose P21 as a time point because at that age the chronic mice had received more than one TNF dose, but the intestine was still immature (as opposed to the P28 intestine which is considered to be matured). There was no significant difference in TNFR1 levels in either B- or C-TNF groups compared to controls (Figure 6(d)).

### 3.8. Chronic TNF Has Age- and Dose-Dependent Effects on Villus Surface Areas and Epithelial Cell Counts

As chronic TNF (C-TNF) caused developmentally dependent villus blunting, we next investigated its effects on villus surface area and epithelial cell counts. While acute TNF treatment induced decreases in villus area and epithelial counts at P14 and 21 (Figure 2), brief (B-TNF) and chronic (C-TNF) treatments induced significant increases in both area and epithelial cell counts at these ages, suggesting a change in villus architecture from long thin villi to shorter, wider villi in the face of chronic inflammation (equal area with less length). Interestingly, this trend was not seen in P28 mice. Exposure to B-TNF induced a significant increase in villus area but a decrease in epithelial cell numbers, and exposure to C-TNF induced no change in villus area and a decrease in epithelial cell counts. These effects demonstrate a developmental stage-dependent effect of chronic exposure to TNF on the small intestinal architecture (Figures 6(b) and 6(c)) (*P* < 0.0001).

### 3.9. Acute but Not Chronic TNF Induces a TNFR1-Dependent Loss of Paneth Cell Populations

**Figure 7:**
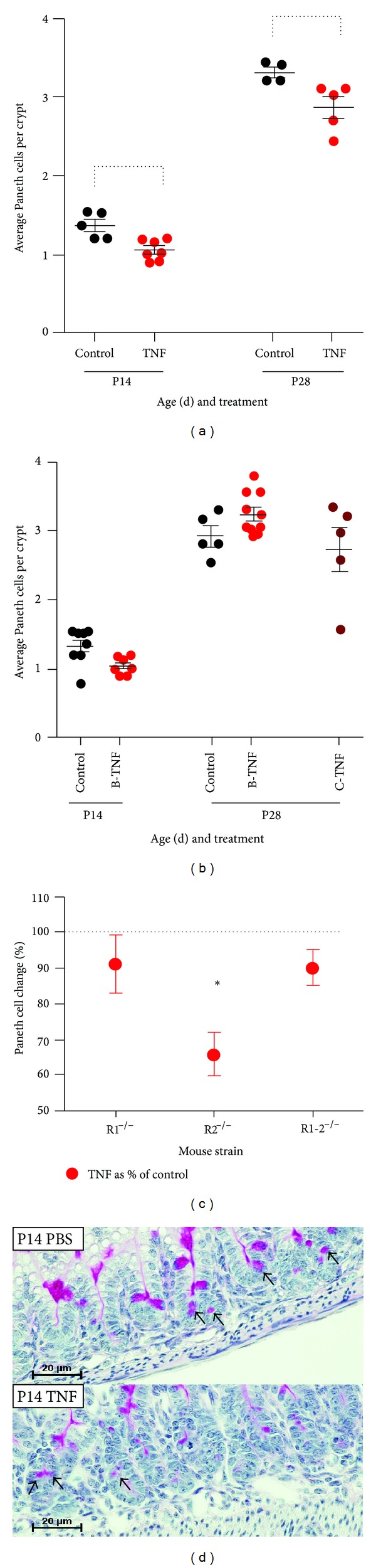
Acute TNF exposure induces loss of Paneth cells in a TNFR1-dependent manner. (a) Mice were treated with acute (injected 8 hours before collection) TNF exposure and granulated Paneth cells per crypt (100 crypts per animal) were counted histologically (*n* = 5 mice per condition; brackets, *P* = 0.002). (b) Mice were treated with brief (B-TNF; single injection with 1-week recovery) or chronic (C-TNF; weekly injections) exposure as above and granulated Paneth cells per crypt were counted; no significant changes were observed. (c) Representative tissue sections are shown. (d) To determine if acute TNF-induced Paneth cell loss was TNFR-dependent, mice lacking one or both TNFR were treated with acute exposure to TNF (*n* = 5, asterisk indicates *P* = 0.035 in R2^−/−^ mice).

Our lab and others have described a key protective role for Paneth cells in the immature intestinal tract [8, 18–20]. TNF has been shown to be important in Paneth cell granule release in adult intestine [21, 22]; however, the effect of either acute or chronic TNF on Paneth cells from immature intestine is less well described. To examine this we treated mice with acute, brief, or chronic TNF exposures as above and quantified the number of granule containing Paneth cells in the ileum compared to controls. Mice were only examined from P14 to P28 as mature Paneth cells do not appear in mice until between P7 and P10 [23, 24]. Acute TNF induced significant loss of granulated Paneth cells in both P14 and P28 mice (Figure 7(a)) (*P* = 0.002). However, there was no difference in Paneth cell numbers in mice treated with brief or chronic treatments (Figure 7(b)). To determine if acute TNF-induced Paneth cell loss was TNFR dependent, we examined the effects of TNF on P14 mice lacking TNFR1, TNFR2, or both. TNF induced significant loss of granulated Paneth cells only in TNFR2^−/−^ mice demonstrating a requirement for TNFR1 (Figure 7(c)) (*P* = 0.035).

## 4. Discussion

Premature infants are routinely subject to intestinal inflammation, both as a result of normal exposure to new environmental factors and through pathologic situations such as intestinal perforations and necrotizing enterocolitis. However, the ramifications of inflammation during development of the intestinal tract are not fully understood. In this study, we describe the normal growth patterns of intestinal villi. We next provide evidence that acute TNF exposure can blunt normal villus height during development. We also demonstrate that chronic TNF exposure can blunt height even more significantly. This blunting occurred through a TNFR1-mediated mechanism. Since TNFR1 expression remains constant through development of the small intestine, TNF-induced villus blunting may be dependent on downstream TNF signaling targets that are differentially expressed during development. Lastly, we show that acute TNFR1-mediated signaling also induces a significant decrease in the number of granule containing Paneth cells. Taken together, these morphologic changes caused by TNF exposure may help to explain why this cytokine plays a prominent role in the development of NEC in the immature intestine.

Our data demonstrate that both acute and chronic TNF exposures induce significant changes in the immature ileum. TNF mediates distinct physiological effects through two separate transmembrane receptors, TNFR1, and TNFR2 [25, 26]. Physiological levels of TNF result in a preferential ligation to TNFR2, which promotes cellular migration, proliferation, and wound healing [27], while higher concentrations of TNF lead to ligation of TNFR1 and subsequent activation of inflammatory responses [25]. Our lab has recently shown that TNF causes TNFR1-dependent, developmental stage-dependent changes in intestinal goblet cell secretion of mucus [13]. Similarly, we demonstrate in these experiments that TNF-induced villus blunting through a TNFR1-dependent, TNFR-2 independent mechanism. The developmental-stage dependent differential effects of TNFR1 are intriguing as protein levels of TNFR1 remain constant from birth through four weeks of life, when the ileum reaches maturation. It was also interesting to note that TNF had little effect on the most immature intestine (P0) despite having similar levels of TNFR1. Our initial hypothesis was that the most immature intestine would show the greatest effects of TNF exposure. However, TNF had no effect on villus length, villus area, or epithelial cell counts. This was similar to our data regarding TNF effects on goblet cells [13] and correlates clinically with a lack of development of NEC during extremely premature ages [28]. These data imply that either an unidentified downstream target of TNFR1 has developmental-stage dependent expression or that another developmental stage-dependent influence acts as a repressor of TNFR1 signaling. Either way, this alteration of TNFR1 signaling may be important, as intestinal inflammation is a common occurrence in premature infants.

The embryogenesis of the gastrointestinal tract is nearly identical in all mammals; however, the stage of development when birth occurs can vary dramatically between species [29]. While human infants have mature intestinal tracts at term birth, mice are born much earlier in the developmental process [29, 30] and do not reach a maturity level equivalent to term human infants until four weeks after birth [1]. Thus, the first four weeks of mouse life reasonably approximate the last half of human fetal development, the stage of development when premature infants are most susceptible to developing NEC. Thus it is important to note that villus length increased during the first four weeks of life as a function of age (Figure 1(a)).

In our experiments to study the effects of chronic inflammation on the intestine, we modeled two types of chronic TNF treatments. Brief dosing examined mice one week following a single TNF treatment, and chronic dosing examined mice one week following the last of multiple weekly TNF treatments. Our data show that both types of chronic exposure to TNF induced villus blunting at P14, P21, and P28. However, it was interesting to note that while chronic exposure to TNF induced loss of villus height at all ages, P14 and P21 mice had significantly increased villus area and epithelial cell counts with this exposure. This suggests a conformation change from thin long villi to shorter, wider villi in the face of chronic inflammation. This may be a compensation mechanism to place the villus epithelial layer closer in proximity to the lamina propria where the majority of adaptive immune cells reside. Interestingly, this trend was not seen in P28 mice, again demonstrating developmental stage-dependent effects of TNF on the small intestinal architecture.

We and others have recently reported a loss of Paneth cells in infants who developed NEC [18, 31], and we have recently developed a novel two-hit model of NEC in mice that utilizes Paneth cell loss to develop injury that is consistent with human disease [18]. In this model, significant injury only occurs following ablation of Paneth cells, suggesting that the injury to, or loss of, the Paneth cell is required to develop NEC. TNF has been shown to induce Paneth cell degranulation in adult mice [22], thus we desired to determine if TNF would have similar effects in the developing intestine. Our data show that acute TNF significantly reduces granule containing Paneth cells in both P14 and P28 mice, and that this reduction is due to a TNFR1-dependent mechanism. Interestingly, chronic exposure to TNF had no effect on the Paneth cell population, suggesting that while Paneth cells are responsive to TNF exposure, the ileum naturally compensates to preserve the Paneth cells in the face of chronic inflammation. These data correspond to additional results from our laboratory that shows decreased expression of lysozyme, a key granular secretion of Paneth cells, in ileal enteroid cultures exposed to TNF [32].

In summary, we have shown that chronic exposure of the immature ileum to TNF induces not only functional changes but architectural changes as well. These findings may have important implications for elucidating the pathophysiology of NEC. Our recently proposed “bottom up” model of NEC hypothesizes that bacteria invade the intestinal tissue principally through the crypts, rather than through the villus tips as previously thought [8]. In this model, TNF-induced blunting caused by feeding-induced or other chronic inflammation could subsequently decrease the distance between the luminal contents of the intestine and the lamina propria. This shortening of distance would greatly increase the ability of bacteria to reach the crypt and infiltrate the intestine. This, along with other effects of TNF such as depletion of the mucous layer and degranulation of Paneth cells, may allow for easier bacterial penetration into the intestinal lamina propria, leading to the inflammatory response and coagulation necrosis characteristic of NEC.
